# Antibacterial Properties of Propolis

**DOI:** 10.3390/molecules24112047

**Published:** 2019-05-29

**Authors:** Izabela Przybyłek, Tomasz M. Karpiński

**Affiliations:** Department of Medical Microbiology, Poznań University of Medical Sciences, Wieniawskiego 3, 61-712 Poznań, Poland; iza.przybylek@gmail.com

**Keywords:** propolis, bee product, antibacterial, *Staphylococcus aureus*, *Escherichia coli*, polyphenols, terpenoids

## Abstract

Researchers are continuing to discover all the properties of propolis due to its complex composition and associated broad spectrum of activities. This review aims to characterize the latest scientific reports in the field of antibacterial activity of this substance. The results of studies on the influence of propolis on more than 600 bacterial strains were analyzed. The greater activity of propolis against Gram-positive bacteria than Gram-negative was confirmed. Moreover, the antimicrobial activity of propolis from different regions of the world was compared. As a result, high activity of propolis from the Middle East was found in relation to both, Gram-positive (*Staphylococcus aureus*) and Gram-negative (*Escherichia coli*) strains. Simultaneously, the lowest activity was demonstrated for propolis samples from Germany, Ireland and Korea.

## 1. Introduction

Propolis is a mixture of substances used by bees to defend the hive. This protection concerns filling cavities in the walls of the hive, reducing the entrance during cold days, and also mummifying the demanded intruders, thus preventing their decay [1]. This explains why propolis is also known as bee glue [2]. The word, propolis, is Greek in origin and means at the entrance to the city [3,4]. Discussions on the origin of propolis have been continuing since ancient times. Doubts have arisen about whether its origin comes from plants or bees. Nowadays, with the development of analytical techniques, the approximate composition of propolis and the factors influencing it are known [1].

Bees collect resins from buds, exudates and other parts of plants, mix them with their own salivary enzymes and beeswax which creates propolis [5,6]. The different continents, regions and plant species used to produce propolis make its composition different from each other. Even though propolis has a different chemical composition, it has similar activities such as antibacterial, antifungal, antiviral, antiparasitic, anti-inflammatory, antiproliferative and antioxidant [7,8,9,10].

In Central Europe, including Poland, bees collect secretion from buds of poplar (*Populus* spp.), alder (*Alnus* spp.). Different poplar species are also a source of resin in other European countries, i.e., Bulgaria, Hungary, Albania and England as well as in temperate zone countries (USA, Mongolia, West Asia and North Africa). Birch (*Betula* spp.) is a source of propolis in Northern Europe, e.g., North-European part of Russia. Bees produce propolis also from buds of willow (*Salix* spp.), oak (*Quercus* spp.), ash (*Fraxinus* spp.), chestnut tree (*Aesculus* spp.) and bark from coniferous trees, such as spruce (*Picea* spp.), fir (*Abies* spp.) or pine (*Pinus* spp.). In tropical countries, bees use the secretions of such plants as *Xanthorrhoea* (Australia), *Acacia* (North Africa), *Plumeria* (Hawaii), *Clusia* (Central America) and *Baccharis*, *Araucaria*, *Eucalyptus* (Brazil) [1,2,11,12,13].

Brazil is the leading country in research on products of bees, including propolis [14]. In Brazil, many types of propolis are distinguished because of their botanical origin. In this climate, bees collect propolis throughout the year. Bueno-Silva et al., in their study, analyzed one of the most common type of Brazilian propolis, referred to as red and whose primary plant source is *Dalbergia ecastophyllum*. The effect of the time of the collection of propolis, its chemical composition and antibacterial activity was examined. Seasonal variability between the concentration of vestitol, neovestitol and isoliquiritigenin was observed. The highest content of these ingredients and antibacterial activity was recorded during the rainy season (the period from January to May) [15].

Another type of propolis comes from stingless bees (e.g., *Melipona mondury*, *M. scutellaris*) and it is called geopropolis. It is very similar to propolis produced by bees belonging to *Apis* spp. in both composition and biological activity [16,17]. Torres et al. in their study compared two ethanolic extracts of propolis (EEP) collected from a stingless species of bees, *Melipona quadrifasciata* and *Tetragonisca angustula*. The study showed the more significant activity of geopropolis extracts against Gram-positive bacteria (*Staphylococcus aureus* MSSA and MRSA, *Enterococcus faecalis*) than Gram-negative (*Klebsiella pneumoniae*, *Escherichia coli*). From the two geopropolis which were analyzed, the *Melipona* species was more effective [18].

Raw propolis cannot be used directly in analysis or treatment. First, it must be extracted in order to dissolve and release the most active ingredients. The following solvents are used as the extractants: ethanol, methanol, water, hexane, acetone, dichloromethane and chloroform. Extracts contain approximately 70% concentration of propolis [19,20]. In terms of antibacterial activity, the content of substances such as flavonoids and phenolic compounds is important [21,22,23]. However, depending on the solvent used, different biological activity is found. Devequi-Nunes et al. found approximately two times higher concentrations of phenolic compounds in ethanolic extracts of brown, green and red propolis than in extracts obtained by supercritical extraction. At the same time, the levels of flavonoids were higher in ethanolic extracts of green and red propolis and lower in brown propolis, compared to supercritical extraction [24]. Wieczyńska et al. in their studies found a stronger antimicrobial activity of ethanolic extracts from Polish propolis than hexane extracts [25].

The aim of this review is to show the main active substances of propolis and antibacterial activity of this bee product.

## 2. Chemical Compounds of Propolis

**Figure 1:**
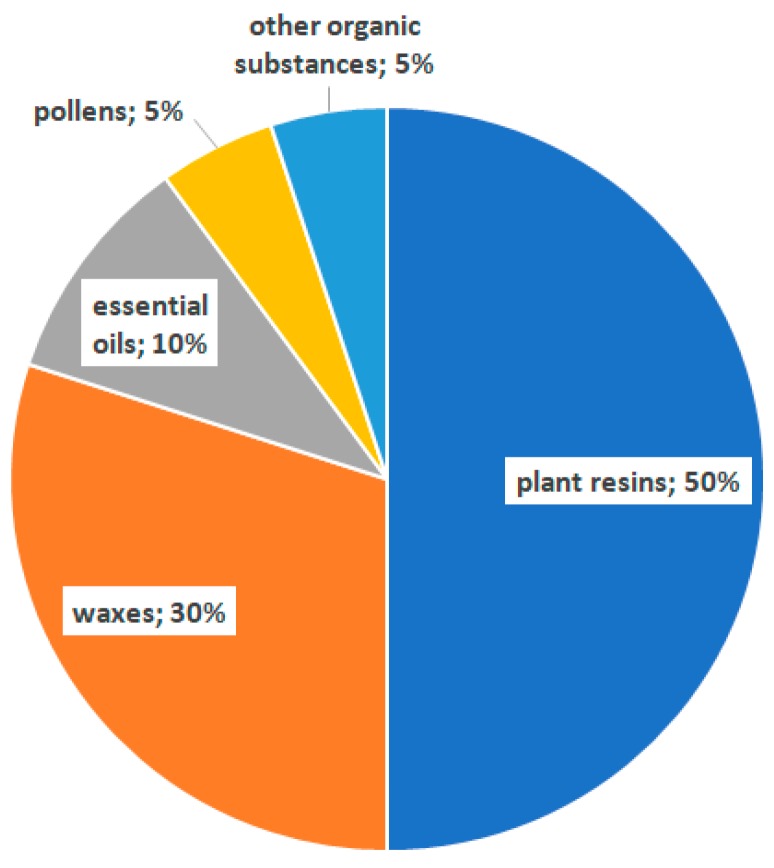
Composition of propolis.

The chemical composition of propolis is closely related to the resins and balsams of plant sources used to produce it. Along with the progress of research, more than 300 chemical components of propolis have been identified. The main groups of chemical compounds found to be present in propolis except resins are waxes, polyphenols (phenolic acids, flavonoids) and terpenoids (Figure 1).

**Figure 2:**
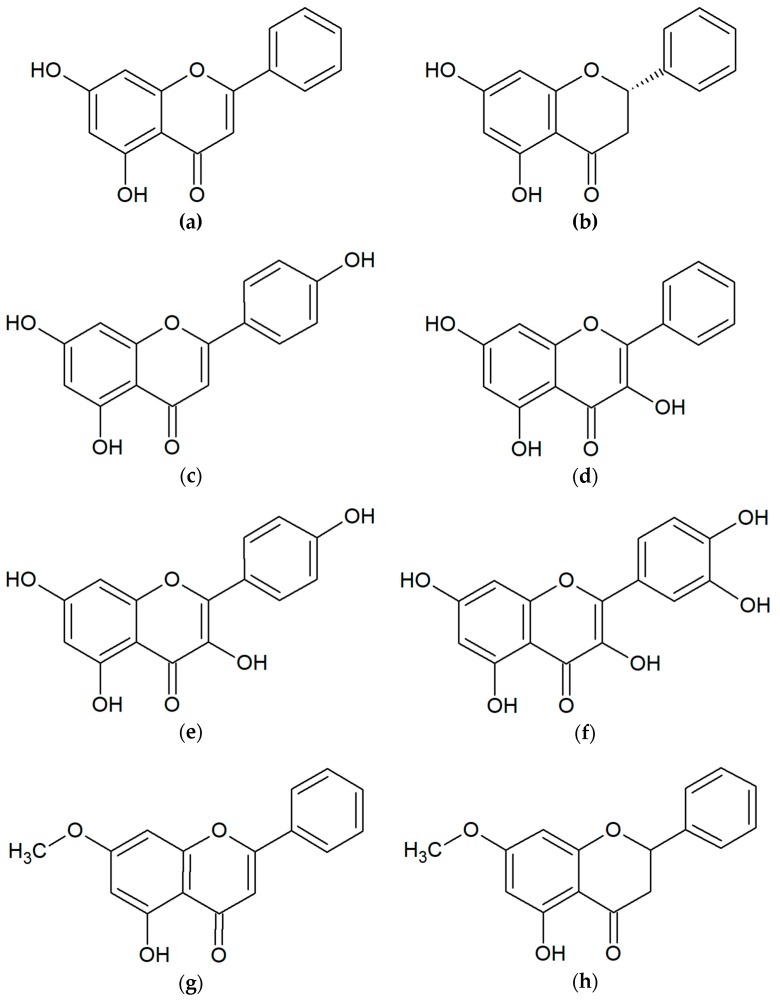
Flavonoids found in propolis: (**a**) chrysin; (**b**) pinocembrin; (**c**) apigenin; (**d**) galangin; (**e**) kaempferol; (**f**) quercetin; (**g**) tectochrysin; (**h**) pinostrobin.

**Figure 3:**
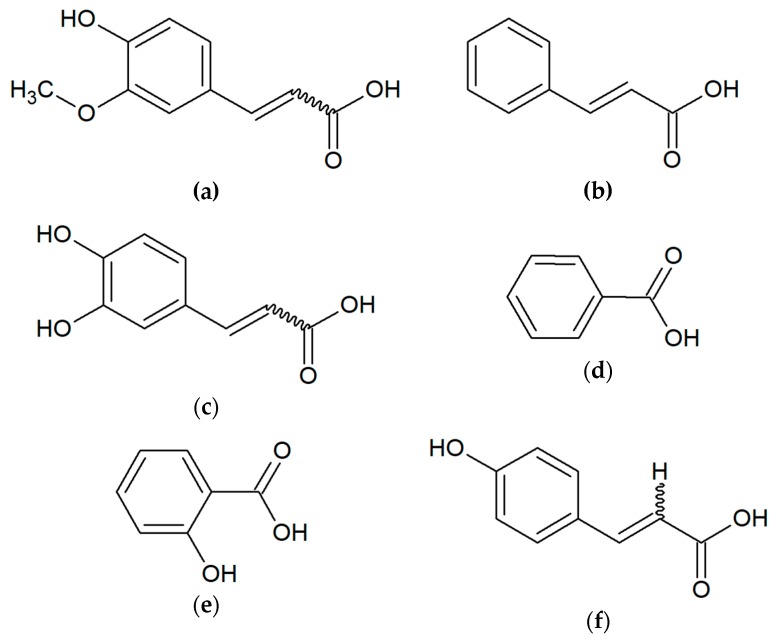
Aromatic acids present in propolis: (**a**) ferulic acid; (**b**) cinnamic acid; (**c**) caffeic acid; (**d**) benzoic acid; (**e**) salicylic acid; (**f**) *p*-cumaric acid.

**Figure 4:**
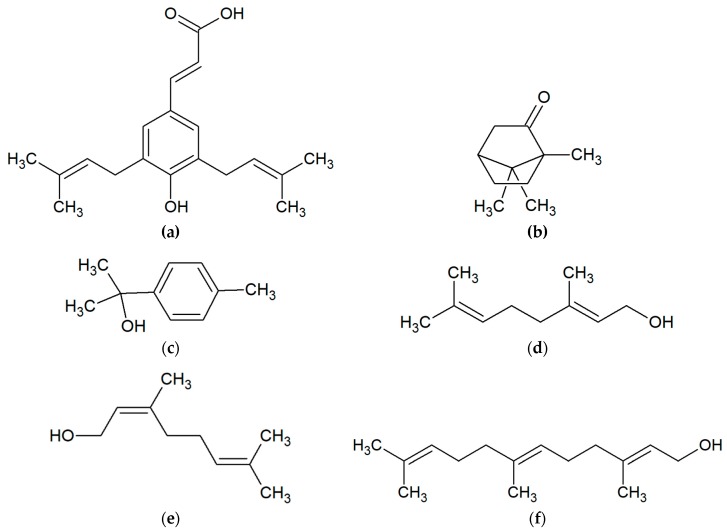
Substances responsible for characteristic fragrance of propolis: (**a**) phenolic compound artepillin C; terpenes: (**b**) camphor; (**c**) terpineol; (**d**) geraniol; (**e**) nerol; (**f**) farnesol.

Polyphenols and terpenoids are also considered to be the most active [26]. The flavonoid group includes chrysin, pinocembrin, apigenin, galangin, kaempferol, quercetin, tectochrysin, pinostrobin and others (Figure 2). Another critical group of compounds of propolis are aromatic acids, among which the most often occur in ferulic, cinnamic, caffeic, benzoic, salicylic and p-cumaric acids (Figure 3) [27,28,29]. In Polish propolis, the content of flavonoid compounds ranged from 6.2 to 18.8%. Among the flavonoids, the highest amounts were pinocembrin (mean 4.7%), pinobenchin (mean 3.1%), galangin (mean 2.2%) and chrysin (mean 2.1%) [27]. In addition, propolis also includes other phenolic compounds (e.g., artepillin C), and terpenes (terpineol, camphor, geraniol, nerol, farnesol) which are responsible for its characteristic fragrance (Figure 4). In propolis, micro and macroelements (Mn, Fe, Si, Mg, Zn, Se, Ca, K, Na, Cu) and vitamins B1, B2, B6, C and E can be found [1,2,28,29,30,31,32]. This diversity of the chemical composition gives propolis an additional advantage as an antibacterial agent. The combination of many active ingredients and their presence in various proportions prevents the bacterial resistance from occurring [33].

## 3. Antimicrobial Properties of Propolis

**Figure 5:**
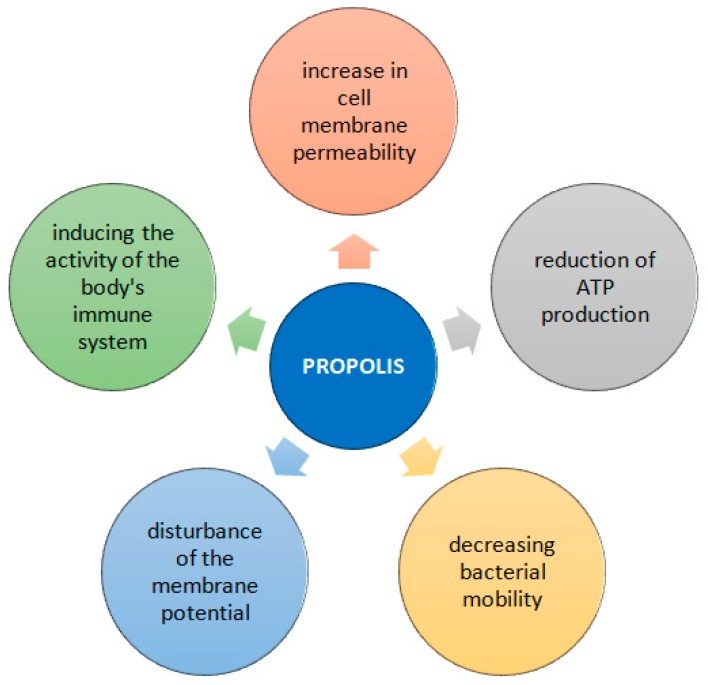
The mechanisms of propolis activity against bacteria.

The antibacterial activity of propolis should be considered on two levels. First, it is connected with the direct action on the microorganism, and the other with stimulation of the immune system resulting in activation of natural defenses of the organism [5]. The analysis of the mechanisms of propolis allow it to infer its effect on the permeability of the cellular membrane of microorganism, disruption of membrane potential and adenosine triphosphate (ATP) production as well as decreasing bacterial mobility (Figure 5). Generally, it is observed that the antimicrobial activity of propolis is higher in relation to Gram-positive than Gram-negative bacteria. This is explained by the species-specific structure of the outer membrane of the Gram-negative bacteria and the production of hydrolytic enzymes which break down the active ingredients of propolis [31,34]. Artepillin C (3,5-diprenyl-*p*-coumaric acid) is one of the numerous phenolic compounds (prenyl derivative of *p*-coumaric acid) found in propolis. Research carried out in Brazil by Veiga et al. shows a higher concentration of artepillin C in ethanolic extracts of propolis compared with hexane extracts. These extracts also showed high antibacterial activity on MRSA *S. aureus* [35]. In studies against anaerobic bacterium *Porphyromonas gingivalis*, it was found that artepillin C has bacteriostatic activity with membrane blebbing [36]. Artepillin C shows additionally anti-inflammatory effects mediated with modulation of NF-kappaB and inhibition of prostaglandin E(2) and nitric oxide [37].

Other prenyl derivatives found in propolis, i.e., 3-prenyl-cinnamic acid allyl ester and 2-dimethyl-8-prenylchromene, also have similar antimicrobial activity [38]. The antimicrobial effect on skin infection-related microbes, such as *S. aureus* also presents kaempferide [39]. The ethanolic extract of propolis containing high concentrations of kaempferide, artepillin-C, drupanin and *p*-coumaric acid showed antioxidant activity and antibacterial against *S. aureus*, *S. saprophyticus*, *Listeria monocytogenes* and *E. faecalis* [40].

Other flavonoids found in propolis are pinocembrin and apigenin. The study conducted by Veloz et al. on Chilean propolis found that antibacterial activity of both of these compounds against *Streptococcus mutans* is higher than activity of polyphenols mixture or even chlorhexidine (MICs = 1.6 µg/mL), with minimum inhibitory concentrations (MICs) 1.4 µg/mL and 1.3 µg/mL, respectively [41]. Several studies have demonstrated the antibacterial activity of isolated pinocembrin against *S. mutans*, *S. sobrinus*, *S. aureus*, *E. faecalis*, *L. monocytogenes*, *Pseudomonas aeruginosa* and *K. pneumoniae* [42,43,44,45]. Isolated apigenin acts against Gram-negative bacteria: *P. aeruginosa*, *K. pneumoniae*, *Salmonella enterica* serotype Typhimurium, *Proteus mirabilis* and *Enterobacter aerogenes* [46]. It was also observed the synergistic antibacterial effect of apigenin with β-lactam antibiotics against methicillin-resistant *S. aureus* (MRSA) [47], and apigenin with ceftazidime against ceftazidime-resistant *Enterobacter cloacae* [48].

Cinnamic acid and its derivatives are a group of aromatic, carboxylic acids commonly found in the plant kingdom. They are present both in green parts of plants and in flowers. Propolis, as a material composed to a large extent of plants secretions, is a rich source of cinnamic acid and esters. Many studies documented the antimicrobial activity of cinnamic acid against *Aeromonas* spp., *Vibrio* spp., *E. coli*, *L. monocytogenes*, *Mycobacterium tuberculosis*, *Bacillus* spp., *Staphylococcus* spp. *Streptococcus pyogenes, Micrococcus flavus*, *P. aeruginosa*, *S. enterica* serotype Typhimurium, *Enterobacter cloacae* and *Yersinia ruckeri* [49,50,51]. Cinnamic acid and its derivatives inhibit bacteria by damaging the cell membrane, inhibiting ATPases, cell division and biofilm formation. Moreover, they had anti-quorum sensing activity [52].

Interestingly, there are many other ingredients of propolis, such as terpenoid lupeol, flavonoids: quercetin, chrysin, kaempferol, fisetin or decanoic acids, i.e., 10-hydroxyl-2-decenoic acid [32]. Some studies are analyzing the antibacterial and anti-inflammatory activity of quercetin, chrysin and kaempferol [45,53,54,55,56,57].

In the research conducted in Korea by Park et al., scientists noticed the effectiveness of lipases in decreasing fatty acids levels in propolis extract. Beeswax and resins are the main components of propolis and both are hydrophobic. The use of fat-degrading enzymes helps to improve the extraction and isolation of active propolis compounds which could make it be used much more widely. The reaction involving lipozyme TL IM increases antimicrobial activity against *Staphylococcus epidermidis* and *Propionibacterium acne* [58].

Kubiliene et al. compared composition and biological activities of propolis extracts prepared with an alternative, nonalcoholic mixture of solvents such as polyethylene glycol 400 (PEG), water and olive oil. They found no significant differences in the total content of phenolic compounds compared with ethanolic extracts. However, the determination of different classes of compounds was not specific. PEG-water solutions presented naringenin, galangin and kaempferol. The addition of PEG connected with exposition for higher temperature enabled extraction of ferulic, caffeic, *p*-coumaric acids, quercetin and artepillin C. Nonalcoholic extract of propolis was prepared by mixing water, oil and PEG and presented similar or higher antimicrobial activity against *S. aureus*, *Bacillus cereus*, *P. aeruginosa* and *K. pneumoniae* then EEP [59].

Recently, the evaluation of the antibacterial activity of propolis extracts is based on the determination of total phenolics content (TP) and flavonoids (FP). In their study, Bridi et al. found that TP and FP tests do not always adequately reflect antimicrobial activity in vitro. The results of the TP in the samples with the highest and the lowest content were directly proportional to the content of flavonoids and antioxidant properties. However, they were not unambiguous in the case of antibacterial activity. It is suggested that other tests, e.g., ORAC (Oxygen Radical Absorbance Capacity) and antimicrobial tests, should be considered in setting international quality standards for propolis [60].

Assuming that propolis and products of other bees have antibacterial activity, their combination should intensify this effect. Al-Waili et al. examined such a dependence on the combination of propolis and honey collected in Saudi Arabia and Egypt. The results of this study confirm that the combination of propolis and honey enhances their antimicrobial effect (for *S. aureus* and *E. coli*). This effect was significant for Saudi propolis (almost two times lowering the MIC value). Egyptian raw materials showed much less activity [61].

There is limited research on the action of propolis on the anaerobic bacteria. However, this research indicates a high activity of this bee product against *Clostridium*, *Bacteroides*, *Porphyromonas*, *Prevotella*, *Fusobacterium*, *Actinomyces* and *Propionibacterium* species [62,63,64,65]. In Polish studies, anaerobic bacteria of the *Fusobacterium* genus were the most susceptible to low concentrations (0.01–0.06 mg/mL) of ethanolic extract from propolis (EEP). However, bacteria of the genus *Actinomyces*, *Bacteroides*, *Clostridium*, *Peptococcus*, *Peptostreptococcus* and *Propionibacterium* were sensitive to EEP in high concentrations (1–3 mg/mL) [66,67,68].

## 4. Data Analysis

The minimal inhibitory concentration (MIC) is the lowest concentration of an antimicrobial agent in which no growth was observed of a microorganism in an agar or broth dilution susceptibility test [69]. This study included scientific publications specifying the MIC for extracts of propolis using the broth dilution method. The MIC is expressed in micrograms per milliliter [µg/mL].

The analysis of the literature completed for this review included a total of approximately 600 propolis MIC values for different groups of microorganisms. In order to visualize the collected results in the form of arithmetic mean values, microorganisms of less than 3 MIC values were excluded from the analysis. These were *Actinomyces naeslundii* (1 record) [16], *B. cereus* (1) [70], *Bifidobacterium bifidum* (1), *B. infantis* (2), *B. longum* (1), *Clostridium butyricum* (2), *C. paraputrificum* (1), *C. perfringens* (1) [71], *L. monocytogenes* (2), and *P. gingivalis* (1) [36].

The ethanol extract of propolis (EEP) was most commonly used in the analyzes, i.e., in 324 records. Methanol (MeEP) was used as the extractant at a similar frequency (209 records). The non-alcoholic extracts are much less frequently analyzed. In such cases, the solvents were water (WEP) in 19 records, dimethyl sulfoxide (DMSO) in 12 records, dichloromethane (DCM) in 10 records, and hexane and supercritical fluid (SCEP) in six records.

**Figure 6:**
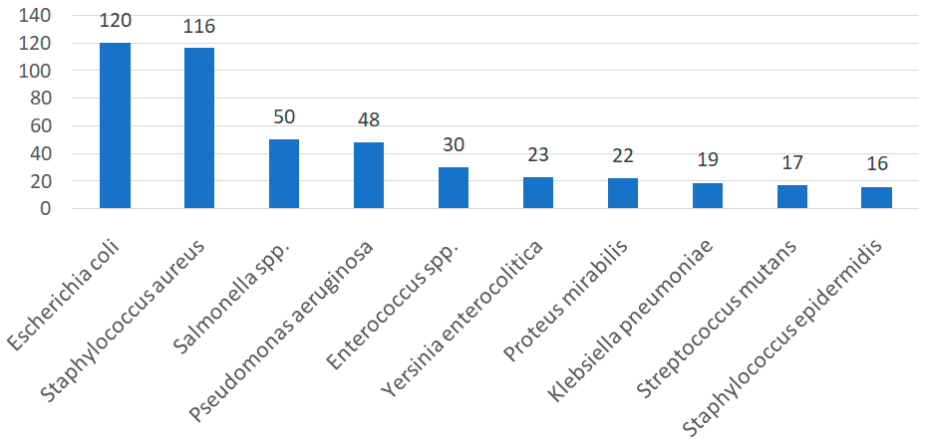
A number of studies with the ten most-analyzed bacterial strains in terms of sensitivity to propolis extracts.

The antibacterial activity of propolis is most often tested on *E. coli*, *S. aureus*, *Salmonella* spp. and *P. aeruginosa*. The ten most frequently analyzed bacteria are shown in Figure 6.

The analysis of the average MIC values for propolis extracts confirmed their higher efficacy against Gram-positive than Gram-negative bacteria. The MIC values for EEP were 117–1840 µg/mL for the first group and 34–5000 µg/mL for the second group of bacteria. All data are shown in Table 1 for Gram-positive bacteria and Table 2 for Gram-negative bacteria.

It is best to compare the effect of propolis (EEP) on the basis of geographical origin for *S. aureus* as a representative of Gram-positive bacteria and *E. coli* representing Gram-negative strains. This is the largest number of available tests on the listed species.

In *S. aureus,* the highest activity was observed for EEP from Turkey, Taiwan and Oman with MIC values of 8, 10 and 81 µg/mL, respectively. The lowest activity was observed for samples of propolis from Chile, Australia and Germany. The MIC values for EEP were 1445, 1200 and 750 µg/mL, respectively. Against *E. coli,* the most active were ethanol extracts of propolis obtained from Turkey, Oman and Slovakia, with MICs of 116, 302 and 510 µg/mL, respectively. At the end, with the lowest activity, there were propolis samples from Germany, Korea and Ireland with MIC values between 1200–5000 µg/mL. Interestingly, Brazil, despite having the largest amount of research on propolis, was in the middle for both Gram-positive and Gram-negative bacteria. The results for all analyzed bacterial species are presented in Table 3 and Table 4.

The most active in both cases were samples of propolis collected in Turkey and Oman. Both countries can be included in the Middle East countries, famous for the trade in fragrances. The same plants are probably a source for bees for the production of propolis. It would also be interesting to analyze the impact of the degree of urbanization of the country on the quality and biological activity of propolis. However, research on this topic is unavailable.

## 5. Conclusions

Propolis is a significant antimicrobial bee product. It acts both against Gram-positive and Gram-negative, as well as aerobic and anaerobic bacteria. The activity of propolis depends on chemical composition and is different in individual countries.

## Figures and Tables

**Table 1:** The activity of different propolis’ extracts against Gram-positive bacteria.

Bacteria	Solvent	Average (min.–max.) MIC Value µg/mL	Ref.
*Staphylococcus aureus*	DCM	364 (16–950)	[72,73]
	DMSO	930 (50–950)	[6,74]
	EEP	457 (8–3100)	[16,60,70,72,74,75,76,77,78,79,80,81,82,83,84,85,86,87,88]
	Hexan	258 (16–500)	[72]
	MeEP	266 (63–1000)	[89]
	WEP	883 (565–1200)	[86]
*Staphylococcus epidermidis*	DCM	900	[73]
	EEP	345 (8–1135)	[75,76,82,90]
*Streptococcus mutans*	EEP	511 (4–4025)	[16,75,76,91,92,93]
*Streptococcus “viridans”*	EEP	682 (150–1370)	[76]
*Streptococcus pyogenes*	EEP	534 (80–1556)	[60,70,86,94]
	WEP	1078 (600–1556)	[86]
*Streptococcus pneumoniae*	EEP	153 (80–300)	[86]
	WEP	1003 (600–1556)	[86]
*Streptococcus oralis*	EEP	167 (100–300)	[86]
	WEP	1070 (940–1200)	[86]
*Streptococcus agalactiae*	EEP	333 (100–600)	[86]
	WEP	2150 (600–3693)	[86]
*Streptococcus sobrinus*	EEP	5 (2–8)	[75]
*Enterococcus* spp.	DMSO	1600	[74]
	EEP	544 (2–1600)	[16,70,74,75,80,82,85,86,94]
	SCEP	698 (63–1000)	[85]
	WEP	250	[86]
*Micrococcus luteus*	DCM	35 (8–63)	[72]
	EEP	117 (4–400)	[72,75,80]
	Hexan	254 (8–901)	[72]
*Bacillus subtilis*	DCM	39 (16–62.5)	[72]
	EEP	180 (21–300)	[72,86,94]
	Hexan	266 (31–500)	[72]
	WEP	250	[86]
*Clostridium difficile*	EEP	1840	[71]

**Table 2:** The activity of different propolis’ extracts against Gram-negative bacteria.

Strain of bacteria	Solvent	Average (min.–max.) MIC Value µg/mL	Ref.
*Escherichia coli*	DMC	1340	[73]
	DSMO	3648 (3190–4940)	[6,74]
	EEP	784 (16–5000)	[70,71,74,75,76,77,84,86,88,94]
	MeEP	303 (31–1000)	[89]
	WEP	2500	[86]
*Salmonella* spp.	EEP	2962 (32–14700)	[75,86,94]
	MeEP	265 (62–1000)	[89]
	WEP	2500	[86]
*Klebsiella* spp.	DCM	1030	[73]
	EEP	1006 (32–3330)	[70,71,76,82,85,86,94]
	WEP	2067 (1200–2500)	[86]
*Yersinia enterocolitica*	EEP	1633 (1200–2500)	[86]
	MeEP	171 (63–500)	[89]
*Proteus mirabilis*	EEP	1947 (512–3080)	[82,94]
	MeEP	618 (250–1000)	[89]
*Shigella flexneri*	EEP	1133 (300–2500)	[86]
	WEP	2500	[86]
*Enterobacter cloacae*	DMC	1150	[73]
	EEP	1926 (300–5000)	[76,86]
	WEP	2500	[86]
*Enterobacter aerogenes*	EEP	34 (8–64)	[75]
*Pseudomonas aeruginosa*	DCM	1100	[73]
	DSMO	2310 (1560–2810)	[6]
	EEP	1252 (32–7910)	[75,76,82,84,86,94,95]
	MeEP	180 (63–500)	[89]
	WEP	2500	[86]
*Acinetobacter baumannii*	EEP	5000	[86]
*Haemophilus influenzae*	EEP	1433 (600–2500)	[86]
	WEP	2500	[86]
*Campylobacter jejuni*	EEP	256 (170–340)	[70,96]
*Bacteroides fragilis*	EEP	2460 (1840–3700)	[71]
*Burkholderia cepacia*	EEP	2467 (1200–5000)	[86]
	WEP	2500	[86]

**Table 3:** The MIC values for EEP from different geographical origin against Gram-positive bacteria (“-”—no data).

		MIC Value [µg/mL]													Ref.
	Strain	*B. subtilis*	*C. difficile*	*Enterococcus* spp.	*M. luteus*	*S. aureus*	*S. epidermidis*	*S. agalactiae*	*S. mutans*	*S. oralis*	*S. pneumoniae*	*S. pyogenes*	*S. sorbinus*	*S. “viridans”*	
Country															
Australia		-	-	-	-	1200	-	-	-	-	-	-	-	-	[83]
Brazil		134	-	631	258	612	825	-	123	-	-	512	-	-	[72,74,80–82,84,85,93,94]
Bulgaria		-	-	-	-	125	-	-	-	-	-	-	-	-	[77]
Chile		-	-	-	-	1445	-	-	4	-	-	1470	-	-	[60,92]
Czech Republic		300	-	250	-	600	-	300	-	100	80	80	-	-	[86]
Germany		300	-	250	-	750	-	600	-	300	300	600	-	-	[86]
Greece		-	-	-	-	393	296	-	602	-	-	-	-	682	[76]
India		-	-	-	-	500	-	-	-	-	-	-	-	-	[79]
Ireland		80	-	500	-	545	-	100	-	100	80	80	-	-	[86]
Korea		-	1840	-	-	-	-	-	35	-	-	-	-	-	[91]
Morocco		-	-	-	-	360	-	-	-	-	-	-	-	-	[87]
Oman		-	-	-	-	81	-	-	-	-	-	-	-	-	[77]
Poland		-	-	-	-	555	1135	-	-	-	-	-	-	-	[78,90]
Slovakia		170	-	1400	-	255	-	-	-	-	-	1400	-	-	[70]
Taiwan		-	-	-	-	10	-	-	-	-	-	-	-	-	[88]
Turkey		-	-	19	11	8	20	-	42	-	-	-	5	-	[75]

**Table 4:** The MIC values for EEP from different geographical origin against Gram-negative bacteria (“-”—no data).

		MIC Value [µg/mL]														Ref.
	Strain	*A. baumani*	*B. cepacia*	*B. frgilis*	*C. jejuni*	*E. cloacae*	*E. aerogenes*	*E. coli*	*H. influenzae*	*Klebsiella* spp.	*P. mirabilis*	*P. aeruginosa*	*Salmonella* spp.	*S. flexneri*	*Y. enterocolitica*	
Country																
Brazil		-	-	-	-	-	-	571	-	961	1947	2293	512	-	-	[74,82,84,85,94]
Bulgaria		-	-	-	-	-	-	1000	-	-	-	-	-	-	-	[77]
Czech Republic		5000	1200	-	-	5000	-	600	1200	1850	-	1200	5000	600	1200	[86]
Germany		5000	5000	-	-	5000	-	5000	2500	1500	-	2500	5000	2500	2500	[86]
Greece		-	-	-	-	931	-	902	-	894	-	-	-	-	-	[76]
Ireland		5000	1200	-	-	5000	-	1200	600	900	-	600	5000	300	1200	[86]
Italy		-	-	-	260	-	-	-	-	-	-	125	-	-	-	[95,96]
Korea		-	-	2460	-	-	-	1840	-	-	-	-	1470	-	-	[71]
Oman		-	-	-	-	-	-	302	-	-	-	-	-	-	-	[77]
Slovakia		-	-	-	255	-	-	510	-	-	-	-	1140	-	-	[70]
Taiwan		-	-	-	-	-	-	640	-	-	-	-	-	-	-	[88]
Turkey		-	-	-	-	-	34	116	-	-	-	120	72	-	-	[75]
